# Antioxidants: Positive or Negative Actors?

**DOI:** 10.3390/biom8040124

**Published:** 2018-10-25

**Authors:** Bahare Salehi, Miquel Martorell, Jack L. Arbiser, Antoni Sureda, Natália Martins, Pawan Kumar Maurya, Mehdi Sharifi-Rad, Pradeep Kumar, Javad Sharifi-Rad

**Affiliations:** 1 Medical Ethics and Law Research Center, Shahid Beheshti University of Medical Sciences, Tehran 88777539, Iran; bahar.salehi007@gmail.com; 2 Student Research Committee, Shahid Beheshti University of Medical Sciences, Tehran 22439789, Iran; 3 Department of Nutrition and Dietetics, Faculty of Pharmacy, University of Concepcion, Concepcion 4070386, Chile; martorellpons@gmail.com; 4 Department of Dermatology, Emory University School of Medicine, Atlanta Veterans Administration Medical Center, Winship Cancer Institute, Atlanta, GA 30322, USA; 5 Research Group on Community Nutrition and Oxidative Stress (NUCOX) and CIBEROBN (Physiopathology of Obesity and Nutrition CB12/03/30038), University of Balearic Islands, E-07122 Palma de Mallorca, Balearic Islands, Spain; tosugo@hotmail.com; 6 Faculty of Medicine, University of Porto, Alameda Prof. Hernâni Monteiro, 4200-319 Porto, Portugal; 7 Institute for Research and Innovation in Health (i3S), University of Porto, 4200-135 Porto, Portugal; 8 Amity Institute of Biotechnology, Amity University, Uttar Pradesh, Noida 201303, India; kumar.aurya@gmail.com; 9 Zabol Medicinal Plants Research Center, Zabol University of Medical Sciences, Zabol 61615585, Iran; mehdi_sharifirad@yahoo.com; 10 Department of Forestry, North Eastern Regional Institute of Science and Technology, Nirjuli 791109, Arunachal Pradesh, India; 11 Phytochemistry Research Center, Shahid Beheshti University of Medical Sciences, Tehran 11369, Iran; 12 Department of Chemistry, Richardson College for the Environmental Science Complex, The University of Winnipeg, Winnipeg, MB R3B 2G3, Canada

**Keywords:** antioxidants, cancer, inflammation, natural products, reactive species

## Abstract

The term “antioxidant” is one of the most confusing definitions in biological/medical sciences. In chemistry, “antioxidant” is simply conceived “a compound that removes reactive species, mainly those oxygen-derived”, while in a cell context, the conceptual definition of an antioxidant is poorly understood. Indeed, non-clinically recommended antioxidants are often consumed in large amounts by the global population, based on the belief that cancer, inflammation and degenerative diseases are triggered by high oxygen levels (or reactive oxygen species) and that through blocking reactive species production, organic unbalances/disorders can be prevented and/or even treated. The popularity of these chemicals arises in part from the widespread public mistrust of allopathic medicine. In fact, reactive oxygen species play a dual role in dealing with different disorders, since they may contribute to disease onset and/or progression but may also play a key role in disease prevention. Further, the ability of the most commonly used supplements, such as vitamins C, E, selenium, and herbal supplements to decrease pathologic reactive oxygen species is not clearly established. Hence, the present review aims to provide a nuanced understanding of where current knowledge is and where it should go.

## 1. Introduction

Aerobic cells produce reactive oxygen species (ROS) as a metabolic process byproduct. ROS cause macromolecules-oxidative damages when body antioxidant defenses are overwhelmed. Jones redefined oxidative stress as a disruption of redox signaling and control [1]. However, a certain amount of oxidative damage takes place even under normal conditions, besides to be observed, a rise in damage rate with aging and disease processes, since antioxidant and repair mechanisms efficiency decreases [2]. On the other hand, oxidative/nitrosative stress (O and NS) has also been markedly implicated in the pathophysiology of many disorders [2]. Briefly, O and NS are defined as an imbalance between ROS production and ROS neutralizing/counteracting organic ability, through antioxidant and detoxifying mechanisms. Reactive oxygen and nitrogen species (ROS/RNS), such as the superoxide radical (O_2_^−.^), hydrogen peroxide (H_2_O_2_), the hydroxyl radical (^.^OH), nitric oxide (NO) and peroxynitrite (ONOO^−^) are naturally produced by all aerobic organisms and exist in cells in homeostasis with antioxidant molecules and enzymes.

Antioxidants are compounds that inhibit oxidation. Oxidation is a chemical reaction that can produce free radicals, thereby leading to chain reactions that can damage the cells of organisms. Antioxidants, such as thiols or ascorbic acid (vitamin C) end these chain reactions. To balance the oxidative state, plants and animals maintain complex systems of overlapping antioxidants, such as glutathione and enzymes (e.g., catalase and superoxide dismutase), produced internally, or the dietary antioxidants vitamins C and E [3]. Antioxidant defenses induction or endogenous ROS/RNS levels reduction is a rapid and clear oxidative stress indicator. Indeed, ROS/RNS production and accumulation is a common denominator in many disorders and environmental insults, at the same time that can cause serious cell damage leading to physiological dysfunction and cell death in almost all aerobes [4]. Antioxidant therapy has long been investigated as a means of reducing the extent of injury resulting from an ischemic stroke with varying degrees of success [5].

Enzymatic and non-enzymatic antioxidant systems in the body, including superoxide dismutase (SOD), catalase (CAT), glutathione peroxidase (GPX), lipid-soluble vitamin E, carotenes, and water-soluble vitamin C, regulate the balance between ROS and antioxidants. On the other hand, dietary antioxidants, mostly obtained from fruit and vegetable consumption, has also been associated with a great balance between free radicals and antioxidant status, which helps to minimize oxidative stress and reduce the risk of cancer, cardiovascular diseases and aging [6]. Indeed, dietary antioxidants comprise a widespread group of colored molecules responsible for multiple bioactive effects, broadly corresponding to different colors to distinct biomolecules classes. Full consumption advantages and health benefits can also be reached through their mixing since they act synergistically. For example, some antioxidants display a more important role in preventing certain diseases, such as cancer, while other ones work better fighting degenerative diseases. In fact, thousands of antioxidants are present in dietary patterns and that some of them may have stronger antioxidant effects. Cancers induce oxidative stress by changing with progression and the resulting antioxidant status differs from patient to patient. In “early” lung cancers, secondary reactive oxygen species (SOSs) are comparable or inferior to that of healthy people. The role of vitamins varies with gender, type of cancer and metastasis [7].

## 2. Antioxidant: Functional Definition

Antioxidants entered in public’s nutritional vocabulary in the 1990s, at a time when researchers were slowly discovering how oxygen-triggered free radical reactions in the body play a key role in aging-associated chronic diseases [8]. Currently, an antioxidant is defined as any substance able to eliminate ROS and derivatives (RNS, or reactive sulfur species, RSS), directly or indirectly, acting as an antioxidant defense regulator, or reactive species production inhibitor [9]. ROS are a molecules group produced over cellular metabolism, due to mitochondrial oxidases action or by other cellular compartments, being this production raised with mitochondrial damage. RNS and RSS result, respectively, from the reaction between ROS and nitric oxide and thiols [9]. ROS/RNS can cause cell damages by covalent joining with other molecules and by stimulating abnormal cell growth, or even senescence induction, which may lead to persistent cells population that produce inflammatory cytokines at large amounts. According to the common belief, so-called antioxidants may block reactive species production-deleterious effects and, therefore, block aging, inflammation and cancer. Their function can be classified into distinct defense lines, according to their mechanisms of action: (a) preventative agents that suppress new radicals formation (which includes enzymes, such as SOD, CAT and GPX, proteins that bind metals, like ferritin and ceruloplasmin, and minerals such as selenium (Se), copper (Cu), and zinc (Zn)); (b) radical scavenging agents that inhibit chain initiation and/or propagation, which includes glutathione, albumin, vitamins C and E, carotenoids, and flavonoids; (c) repair and de novo enzymes that repair and reconstitute cell membranes, which includes lipases, proteases, DNA repair enzymes, transferases, and methionine-sulfoxide reductases; and (d) adaptation agents that generate appropriate antioxidant enzymes and transfer them to essential site of action [9,10]. Redox balance is essential in healthy cellular microenvironment maintenance.

## 3. Antioxidant Role in Redox Imbalance Prevention: Gaps of Knowledge

**Figure 1:**
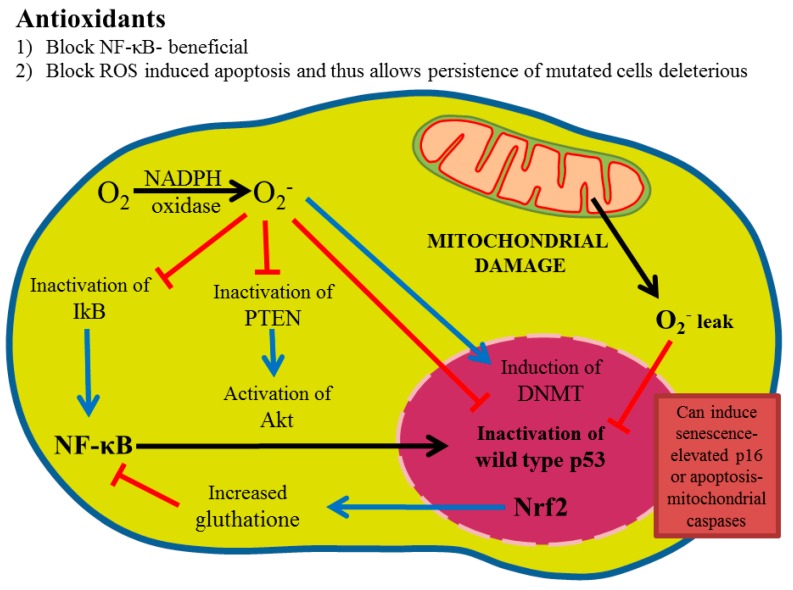
Overview of reactive oxygen signaling. Superoxide, which can arise from NADPH oxidases action or mitochondrial leak, oxidatively inactivate p53, PTEN, and IkB, leading to Akt and NF-κB activation. Reactive oxygen inhibition with NADPH oxidase inhibitors can reverse this phenotype. Glutathione formation through Nrf2 activation can lead to reactive oxygen reduction and, thus, possibly an NF-κB decrease but also may react with chemotherapy and radiation generated species, thus, protecting tumor cells.

Redox imbalance is caused by a balance alteration between ROS and its derivatives and of the cell antioxidant defense system efficiency [10]. Cells and tissues are continuously being exposed to reactive species derived from metabolism or external factors, like smoking, pollution, pesticides, microbes, allergens, and ultraviolet and gamma radiation, which generate free radicals, all of them associated with both aging and other diseases [11]. In addition, reactive species production can overcome body ability to eliminate or to contain them. The antioxidants field has been hampered by the following gaps of knowledge: (1) What are the correct antioxidants doses? There is ample evidence that more is not better and may be worse [12]. Virtually, all so-called antioxidants do not demonstrate a classic dose-response but may have opposite effects with changing doses. (2) How are antioxidants absorbed? Little is known on antioxidants gut microbial metabolism, as well as whether antioxidants affect gut microflora in a pro- or anti-inflammatory way. It is also poorly understood whether antioxidants are absorbed unchanged or metabolized to completely different compounds. One example is ellagic acid, a poorly soluble compound that is metabolized into more soluble metabolites, that can mediate its anti-inflammatory activity [13,14]. (3) Antioxidants naturally occurring may be absorbed as complexes of two or more compounds and complexes may have very different biological activities than isolated compounds alone. (4) Even with ideal absorption and penetration into tumors, tumors may respond with compensatory pathways. For example, ROS inhibition may lead to compensatory mitogen-activated protein (MAP) kinase activation, that could manifest as more rapid tumor growth, despite the fact that these tumors might be deficient in nuclear factor kappa B (NF-κB) activation and more susceptible to chemotherapy or radiation [15] (Figure 1).

## 4. Adverse Effects of Antioxidants

The most popular “antioxidants” forms include vitamins, such as vitamin A (retinol, retinoic acid), vitamin C (L-ascorbic acid, ascorbic acid, ascorbate), vitamin E (α-tocopherol), β-carotene, minerals, like Se, and naturally-occurring polyphenols, each one has a different effect on body cells. Vitamins and β-carotene have conjugated double bonds and key functional groups responsible for their antioxidant role and quality as pigments in several foods, like fruits and vegetables. Below, we briefly summarize the adverse effects of these popular antioxidants, often consumed as supplements at much higher doses than those found in foodstuffs. While their adverse effects are known in the medical community, they are not well-known among the population, who believe that natural products cannot be toxic. Czernichow investigated the effect of antioxidant supplementation for 7.5 years on metabolic syndrome (MetS) incidence and even the epidemiologic association between baseline serum antioxidant concentrations and MetS prospective risk [16]. No beneficial effects of antioxidant supplementation were observed in a generally well-nourished population. Baseline serum antioxidant concentrations of β-carotene and vitamin C, however, were negatively associated with MetS risk. Baseline serum zinc concentrations were positively associated with the risk of developing MetS [16]. Park [17] found that there was no association between dietary intakes of vitamins A, C, and E and colon cancer risk in this pooled analysis of thirteen prospective cohort studies. However, total vitamins A, C, and E intakes were each one inversely associated with colon cancer risk. Multivitamin use, particularly in combination with a single vitamin A, C and/or E supplements use, was inversely associated with colon cancer risk. A low dietary intake of antioxidant vitamins and minerals raises the incidence of cardiovascular diseases and cancer [17]. After 7.5 years, low-dose antioxidant supplementation lowered total cancer incidence and all-cause mortality in men but not in women. In fact, supplementation may be effective in men only because of their low basal status of certain antioxidants, especially of β-carotene [18].

Researchers reported the existence of increased teratogenicity risk or birth defects among babies born from women who took more than 10,000 IU vitamin A per day in the form of supplements [19]. Indeed, excessive dietary vitamin A intake has been associated with birth defects in humans in several studies reported in past years [20,21,22]. A controlled clinical trial found that people who took 25,000 IU of vitamin A per day for a median of 3.8 years had 11% increase in triglycerides, 3% increase in total cholesterol and 1% decrease in high-density lipoprotein (HDL) cholesterol, unlike those who did not take vitamin A [23]. In a recent case report, a 4-year-old boy presented several bone pains due to vitamin A toxicity (600,000 IU every day for more than 3 months) [2]. In fact, it has been reported that excessive vitamin A intake can accelerate bone loss and risk of hip fracture, possibly due to vitamin A-induced osteoclasts stimulation, besides it inhibits new bone formation, increasing osteoporosis risk [24]. 

On the other hand, vitamin C can be metabolized to oxalate and might increase kidney oxalate excretion. Several studies suggest that vitamin C supplements may increase urinary oxalate concentrations, doubling the risk of calcium oxalate kidney stones [25,26,27]. A study defined that high vitamin C intake from supplements is associated with a rise in cardiovascular disease mortality in postmenopausal women with diabetes but this has never been confirmed [28]. Theoretically, vitamin C may cause too much iron absorption but this is likely to be significant only in persons who have high iron stores or in patients with iron overload, such as hereditary hemochromatosis, where an increasing iron toxicity risk may exist [29]. Pavlotou [30] evaluated free oxygen radicals (FORT) and free oxygen radicals defense (FORD) levels in patients with newly diagnosed type 2 DM patients. The authors found that FORT levels were increased in diabetic patients compared to controls; however, FORD levels were lower in diabetic patients compared to controls.

A study reported that dietary vitamin E supplementation significantly increases prostate cancer risk among healthy men [31]. A meta-analysis renders more evidence of vitamin E adverse effects on stroke subtypes. Indeed, the study defined a 22% increased hemorrhagic stroke risk and a 10% decreased ischemic stroke risk with vitamin E supplementation, although the absolute effects are minor [32]. Still, a study underlined that 22–30 mg/day of vitamin E in human pregnancy may be associated with birth weight decrease [33].

Scientists have reported that β-carotene supplementation (and not the intake of vegetables rich in β-carotene) has actually increased the risk of death from lung cancer or heart disease in smokers, rather than reducing cancer incidence [34,35,36,37]. In fact, we believe that other antioxidant side effects may not be reported and other ones will be discovered in the future. Nonetheless, the studies performed so far have severely impaired the reputation of antioxidants in general. However, in an ideal world, we could go back to this study, through examining tissues pre- and post-treatment, and determine whether interventions had a signaling effect in the tumors. Based on previous studies of ebselen, an organoselenium compound with broad antioxidant properties [38], it is likely that ROS reduction in tumors, for instance in Burkitts lymphoma, results in MAP kinase signaling activation and an increased tumor growth rate [15]. In addition, more rapidly growing tumors may be more susceptible to subsequent chemotherapy and radiation. Thus, the current state of affairs suggests that the most common antioxidants are, at best, ineffective and potentially harmful.

## 5. Are Antioxidants Benefits More Apparent Than Real?

Part of the problem regarding antioxidants is trying to impose an antioxidant chemical definition on a biochemical system. Ancient man used carbon as an antioxidant to reduce iron ore to iron, through removing oxygen in iron ore with carbon and driving off oxygen as carbon dioxide. This definition, while applicable to metal refining, does not consider the complexity of biological systems. For example, nuclear factor erythroid 2-like 2 (Nrf2) transcription is one of the most studied antioxidant systems in biology, being normally Nrf2 complexed with Kelch-like ECH-associated protein 1 (Keap 1). So, cells treated with Nrf2 inducers leads to Nrf2 nuclear translocation [39,40]. Many Nrf2 inducers bind to Nrf2 through a Michael addition, oxidizing Nrf2 sulfhydryl groups. Thus, many Nrf2 inducers are oxidants and, therefore, an antioxidant response is an oxidant response [41,42,43]. Furthermore, reduced glutathione, the main small antioxidant molecule in mammalian cells, is a product of several Nrf2downstream target genes, counterbalancing mitochondrial ROS production [44]. Nonetheless, there is an open question whether the antioxidant effect of glutathione synthesis exerts antioxidant activity beyond correcting original oxidative stressor or instead exerts multiple functions, beyond reducing intracellular milieu maintenance [44]. Finally, in glutathione, free sulfhydryl groups (-SH groups) could exert antioxidant effects but, at the same time, bind to chemotherapy reactive intermediates and block effective tumor cells killing [45,46,47]. This fact may explain why glutathione induction/supplementation has not been an as effective strategy against cancer as originally implied. Tumors with mutant p53 demonstrate high Nrf2 activation levels and this could protect tumor cells against reactive adduct induced DNA damage [48].

**Figure 2:**
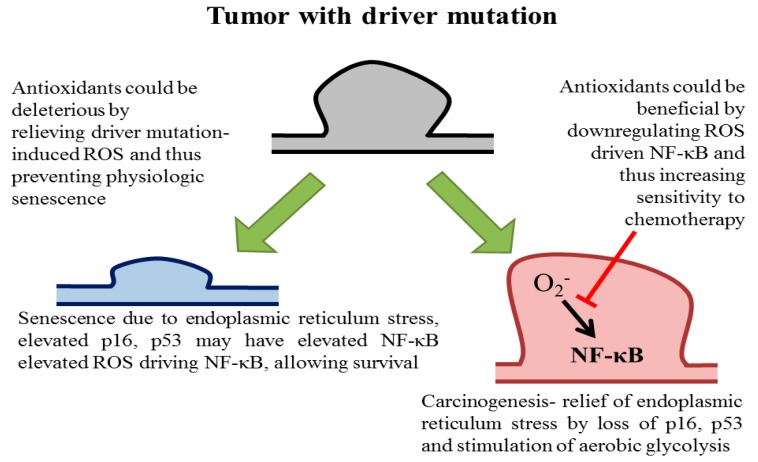
Potential outcomes of antioxidants at differing tumorigenesis stages. The presence of a driver mutation in a primary cell leads to reactive oxygen-mediated endoplasmic reticulum (ER) stress. Normally, this could lead to senescence, in which P16ink4a and FOXO4 senescence markers are elevated, as well as NF-κB activation. This leads to a persistent senescent phenotype which cannot re-enter the cell cycle. On the other hand, ER stress relief with an antioxidant, or tumor suppressor (i.e., p16ink4a) loss leads to clonal cells expansion with driver mutations. This may lead to carcinogenesis. In advanced tumors, NF-κB might be activated by ROS and a ROS blockade may lead to NF-κB activation decrease and chemotherapy and radiation sensitivities increase.

In a recent study, Nrf2 was found to be induced by oncogenic Ras and Nrf2 loss was found to be protective in transgenic models of carcinogenesis. In this study, endogenous oncogenic Ras was found to decrease ROS through Nrf2induction, while exogenous oncogenic Ras overexpression increases ROS levels. Indeed, Nrf2 loss decreased preneoplastic pancreatic cells expression and features of senescence were observed in Nrf2 deficient tumor cells [49]. Thus, these studies suggest that ROS may play a physiologic role in preventing preneoplastic cells propagation with Ras driver mutations (Figure 2).

Treatment with antioxidant N-acetyl cysteine relieved the block, which is consistent with: (a) Cells with oncogenic driver mutations exhibit endoplasmic reticulum stress, which might be in part mediated by high ROS levels, and therefore high ROS levels relief may allow preneoplastic cells proliferation and frankly malignant cells conversion and (b) other observations that antioxidants paradoxically promote cancer [9,12,48,50,51,52].

ROS have distinct tumor-promoting effects, which include DNA methyltransferase 1 (DNMT) induction [53,54], oxidatively tumor suppressors p53 inactivation, kB (IkB), phosphatase and tensin homolog (PTEN) inhibition [55]. This leads to protumorigenic NF-κB and Akt activation and, therefore, treatment with a superoxide production inhibitor (NADPH oxidase inhibitor) leads to p53 reactivation, and NF-κB and Akt inactivation. This would be beneficial as NF-κB and Akt inhibition primes to increase chemotherapy and radiation susceptibility through the downregulation of target genes, such as multidrug resistance protein 1 (Mdr1) and DNA repair genes. On the other hand, sulfur supplementation in terms of increasing reduced glutathione, or Nrf2 induction, could have deleterious effects on chemotherapy and radiation by protecting tumor DNA.

As it has been exposed, supplemental antioxidant administration during chemotherapy and radiation therapy is currently controversial [9,10,12,56,57]. So far, it has been difficult to determine which antioxidants may exert a beneficial impact on cancer treatment outcomes or which may contribute to treatment adverse effects’ amelioration [9,57]. For this reason, during chemotherapy and radiation therapy, an antioxidant prescription is confusing and consequently, this should consider the type of cancer, background and state of patient, antitumor therapy, drugs mechanism of action and drugs used in treatment, as also the antioxidant type and dosage [9,57].

## 6. Redox Imbalance Positive and Antioxidant Effect Negative

It is now well-established that reactive species and a basal redox imbalance level are essential for cell survival [58]. Concomitantly, it is also well-known that while severe redox imbalance often leads to widespread oxidative and nitrosative damage and cell death, a moderate redox imbalance level, induced by wide stressors variety, can yield great beneficial effects on adaptive cellular responses, such increased endogenous antioxidant defense systems levels [12,58].

Hormesis was defined as a cellular adaptive response to stressors that results in a biphasic dose-response relationship, such that low-dose stimulation results in a beneficial adaptation, whereas a high-dose results in a toxic effect [59]. In this context, low ROS doses, produced during exercise training, are required for the exercise-induced training response in skeletal muscle. Thus, ROS are required for exercise adaptive response and are essential to enhance sports performance [59]. In this sense, low cell stressor doses, such as chemicals, toxins, radiation and moderate exercise results in an adaptive response, increase the antioxidant capacity of cells. In fact, exercise itself can be considered an antioxidant, since training increases classical antioxidant enzymes expression, like SOD and GPX, while, in general, antioxidant supplements may not be a good strategy when training because they eliminate ROS production that acts to stimulate endogenous antioxidant enzymes [60]. Moreover, mitochondriogenesis is regulated by many redox-sensitive enzymes, involving MAP kinases, NF-κB, p53, heat shock factor, peroxisome proliferator-activated receptor gamma coactivator 1-alpha (PGC-1α), and others involved in modulating muscle adaptation to muscle [60].

On the other hand, it is believed that antioxidants can prevent cancer development affecting the cell cycle, inflammation, tumor proliferation and invasiveness, apoptosis and detox mechanisms. So, the antitumor effects of several antioxidants (i.e., catechins, isoflavones, lignans, flavanones, resveratrol, ellagic acid, quercetin, and curcumin) have been extensively studied [9]. However, antioxidant supplementation may block endogenous antioxidants raise and other cell adaptation mechanisms, such as better energetic metabolism. Indeed, a basal redox imbalance level is crucial for cell adaptation. The question is what reactive species concentration range is beneficial and what is harmful? In this context, the correlation between cigarette smoke and lung-cancer is well-established but will there be a beneficial low-dose? (Hardly an ethics committee would approve this kind of experimental study).

## 7. What Antioxidants Can Do to Improve Health or What They Cannot Do?

Despite billions of dollars spent on antioxidant supplements yearly, the modification of a chronic disease course remains elusive. Numerous agents have demonstrated chemopreventive effects in murine models but these have not yet been translated to human diseases. Currently, we have enough clinical evidence that antioxidants available as supplements have at best little value in preventing or modifying a chronic disease course. Inherent differences in rodents versus human’s biology, high-dose treatment in rodents and non-delivery of human tissues are probably behind the current failure record. However, there is room for optimism but it can only come from a sophisticated understanding of chronic disease biology. In the case of cancer, currently available antioxidants could be harmful in preventing physiologic senescence in patients who have driver mutations lesions predominance. Physiologic senescence prevention could expand mutated cells population that can undergo carcinogenesis. However, in advanced tumors, ROS downregulation agents inducing NF-κB could be useful in chemotherapy and radiation combination to trigger tumor cell apoptosis.

There is nuance in this as well, as an agent that increases sulfhydryl’s, either through exogenous sulfhydryl supply or Nrf2 induction, could protect tumor DNA and inactivate chemotherapeutic agents. What is needed is a “smart antioxidant”, i.e., one that could cause a redox imbalance in tumor cells but not in normal tissues. A potential category of compounds is sirtuin 3 activators. Indeed, sirtuin 3 is a major mitochondrial NAD^+^-dependent deacetylase that plays a critical role in mitochondrial proteins activation, is involved in energy metabolism, and changes in its expression are associated with excessive ROS production [61]. There is evidence that some polyphenolic compounds found in nature could act as “smart antioxidant” via sirtuin 3 activation [62,63,64]. Such an agent could potentially decrease NF-κB at the same time as not inducing Nrf2. This would tip the balance and could be useful in both oxidative precursor lesions destruction with driver mutations as well as sensitizing advanced cancer to chemotherapy and radiation.

## 8. Conclusions and Upcoming Perspectives

In conclusion, to minimize chronic redox imbalance damages, it is best to follow a balanced and varied diet, including in its composition many grains, legumes, fruits and vegetables of different colors. In addition, healthy lifestyle habits should be included, such as exercise training on a regular basis to avoid obesity, not smoking and reducing alcoholic beverages intake. The intake of antioxidant supplements would only make sense in a case of deficits, trying to normalize their levels, but not as a usual intake. In addition, antioxidants therapeutic usefulness against cancer still has many open fronts that should be investigated in the future.
